# Phenomenal consciousness: its scope and limits

**DOI:** 10.1098/rstb.2024.0306

**Published:** 2025-11-13

**Authors:** Nicholas Humphrey

**Affiliations:** ^1^ Darwin College, Cambridge University, Cambridge, Cambridgeshire, UK

**Keywords:** sentience, consciousness, blindsight, phenomenal self

## Abstract

In the history of life, consciousness of sensory states with ‘phenomenal properties’—the basis of ‘sentience’—is, arguably, a late evolutionary development, which occurred long after conscious access to a ‘global mental workspace’ had become widely established as a strategy for cognitive information processing. In this article, I focus on phenomenal consciousness. I propose a step-by-step sequence by which the mental representation of sensory stimulation could have acquired phenomenal content through small changes in the brain. Also—addressing the question of evolutionary function—I point to the crucial psychological benefits to an animal of having a ‘phenomenally conscious self’. A thread running through the article is the phenomenon of ‘blindsight’, which I take to be a model for the non-phenomenal cognition that characterizes the majority of insentient animal species.

This article is part of the theme issue ‘Evolutionary functions of consciousness’.

## 1. Introduction

The word sentience is on everybody’s lips. The United Kingdom’s government has passed an ‘Animal Sentience Act’ declaring that lobsters and octopuses are ‘legally sentient’. There is a flurry of speculation about sentience in intelligent machines. Respected philosophers suggest the whole universe could be sentient.

If you ask people what they mean by sentience, they tend to avoid your eye. Nonetheless, it is clear from the surrounding discussion that what most people have in mind is some version of *sensory awareness,* as we ourselves experience it. In the words of John Locke, ‘consciousness is the perception of what passes in a Man’s own mind.’ [1, p. 19] But, we tend to be much more impressed by some kinds of mental representation than by others, and the kind that outshines everything else are those representations we call ‘sensations’: the pain of a bee-sting, the smell of coffee, the redness of a cherry. Sensations have a qualitative dimension that philosophers call ‘phenomenality’.

This article is about phenomenal consciousness as a biological phenomenon: its evolutionary history, its functions and its distribution in the animal kingdom. I assume that such a surprising mental faculty can hardly be a mere byproduct of brain complexity: rather, it has evolved because it confers novel adaptive benefits on those animals that possess it. Different species of animals alive today may possess it to varying degrees; but there is a categorical distinction between animals that are phenomenally conscious and those that are not—today’s sentient animals had totally insentient ancestors.

To prepare the ground, and introduce some key theoretical issues, I will begin by discussing my encounter fifty years ago with an animal in whom one aspect of phenomenal consciousness—the capacity to experience sensations of light—had been obliterated by brain surgery.

## 2. Blindsight

The cover of *New Scientist* magazine in 1972 showed a picture of a rhesus monkey with the headline ‘A blind monkey that sees everything’. The monkey, named Helen, was part of a study led by my PhD supervisor, Larry Weiskrantz, in the psychology laboratory in Cambridge. A few years earlier, Weiskrantz had surgically removed the primary visual cortex (V1) at the back of Helen’s brain. When I first met Helen a year after the operation, it seemed that the loss of cortex had, in fact, made her completely blind.

However, something puzzled me. In mammals, there are two main pathways from the eye to the brain: an evolutionarily ancient one—the descendant of the visual system used by fishes, frogs and reptiles—that projects to the optic tectum in the midbrain, and a newer one that projects directly to the cortex. In Helen, the cortical pathway was eliminated, but the ancient visual system was still intact. If a frog can see using the optic tectum, why not Helen?

While Weiskrantz was away at a conference, I took the chance to investigate further. I simply sat with Helen and played with her, offering her treats for any attempt to engage with me by sight. To my delight, she began to respond. Within a few hours, I had her reaching out to take pieces of apple from my hand; within a week, she was reaching out to touch a small flashing light. . . Seven years later, she was running around a complex arena, deftly avoiding obstacles, picking up peanuts from the floor ([2]; electronic supplementary material, video S1).

To anyone who had observed Helen in 1972—and did not know the history—it might have seemed that her eyesight was now quite normal. Yet, could she in fact ’see everything', as the cover of *New Scientist* implied? I myself did not really believe so. I found it hard to tell what was missing. However, my hunch was that Helen herself still *doubted* she could see. She seemed strangely unsure of herself. If she was upset or frightened, her confidence would desert her, and she would stumble about as if in the dark again. The title I gave to the article inside the magazine was ‘Seeing and nothingness’ [3].

We were on the brink of a remarkable discovery. Following on from the findings with Helen, Weiskrantz took a new approach with a human patient. Patient DB had undergone surgery to remove a growth affecting the visual cortex on the left side of his brain. The operation had apparently rendered him blind across the right-half field of vision. DB himself maintained he had no visual awareness in the affected area. Weiskrantz, however, now decided not to take his word for it; and, overcoming his protests, he asked him to guess what he would be seeing *if* he could see. To everyone’s surprise, it turned out he could in fact guess both the location and shape of an object to which he believed he was blind.

DB himself was the most surprised of all. To him, this success in guessing seemed quite unreasonable. So far as he was concerned, *he* was not the source of his perceptual judgments; he did not *own* his ability to see. Weiskrantz named this capacity for vision in the absence of sensation ‘blindsight’ [4].

Blindsight is now a well-established clinical phenomenon, but when first discovered, it seemed theoretically quite shocking. A patient with blindsight has no visual sensation of the light arriving at his eye. Yet, he is still able to use the visual information to perceive the existence of objects out there in the world. No one had ever expected there could be any such dissociation between sensation and perception.

## 3. The puzzle of sensations

As I ruminated on the implications of this for understanding consciousness, I found myself doing a double-take. Rather than asking about the absence of sensation in blindsight, shouldn’t we asking about the presence of sensation in normal sight? If blindsight is seeing and nothingness, normal sight is seeing and somethingness. Surely, it is the nature of this *something* that we have to explain.

Why do sensations feel the way they do? Not only in the case of vision, but across all sensory modalities: our experience of the redness of red; the saltiness of salt; the paininess of pain. What does the extra phenomenal dimension amount to, and what is the use of it?

Sensations, to be clear, have a different remit from perceptions. Both are forms of mental representation; they are ideas generated by the brain. However, they represent—they are about—very different kinds of things. Perception is about ‘what’s happening out there in the external world’: the apple is red; the rock is hard; the bird is singing. By contrast, sensation is personal, it is about ‘what’s happening to me and how I as a subject evaluate it’: the pain is in my toe and horrible; the sweet taste is on my tongue and sickly; the red light is before my eyes and stirs me up.

It is as if, in having sensations, we are both registering the objective fact of stimulation and expressing our bodily opinion about it, and, as I will explain shortly, I think that is just what we are doing. However, why do it *this* way? What makes the subjective present created by sensations seem so rich and deep, as if we are living in ‘thick time’? What can the artist Kandinsky mean when he writes: ‘Colour is a power which directly influences the soul. Colour is the keyboard, the eyes are the hammers, the soul is the piano with many strings’ [5, p. 25]? Why indeed do we use the strange expression ‘it’s like something’ to experience sensations? Is it because sensations are like something they can't really be?

This is the ‘hard problem’ of consciousness. How can a physical brain underwrite the extra-physical properties of phenomenal experience? As the neuroscientist Christof Koch once wrote to me, it’s such a mystery ‘It seems to call for God’.

For fifty years, I have been searching for an answer that does not require God.

## 4. Neural identity?

From the start, I have been suspicious of theories that attempt to identify the ‘neural correlate of consciousness’. Many theorists believe that conscious states are *identical* to brain states, and, for them, the obvious and best approach to the problem is to search for brain events that have phenomenal properties built into their physical structure.

This is actually quite an old proposal. Going back to the 1929 Encyclopaedia Britannica entry on ‘Consciousness’, for instance, you could read about the ‘psychonic theory’ [6]:

One theory holds that each atom of the physical body possesses an inherent attribute of consciousness. If each atom, or, in later forms of this theory, each cell of the body emanates its own consciousness, then the ‘self’ must actually consist of an amalgam of all these tiny units of awareness.

Today, of course, the language has moved on. Yet several currently popular theories are near neighbours of the psychonic theory. ‘Integrated information theory’, for example, postulates in Koch’s words that ‘there is a complete one-to-one mapping between any experience and all of its phenomenal distinctions and relations on the one hand, and the causal structure that is identical to it and that is unfolded from its physical substrate on the other hand’ [7, p. 182] .

However, as I see it, this and all such physical identity theories have got off on the wrong foot. They were—and are—attempts to explain how phenomenal properties could be properties of a brain process. Also this rests on a fundamental misunderstanding of what it is we are trying to explain.

## 5. Representation

Sensations, as I wrote a moment ago, are not material entities, they are *ideas*: they are the way the brain *represents* what is happening at our sense organs and how we, as the conscious subject, *feel* about it. This means consciousness science has to explain their properties, not literally as the properties of brain states, but rather as the properties of mind-states dreamed up by the brain. Once we see this to be the task, I think much of the difficulty and mystery falls away.

As with any kind of representation, we can assume that representing sensations amounts to a two-stage process. In the case of seeing red, for example, there will be (i) the physical vehicle that carries the information about how the brain evaluates the light arriving at the eyes, and (ii) the cognitive operation that interprets this as the ‘idea of phenomenal redness’. By analogy, consider a work of literature: say, the novel *Moby Dick*. There will be (i) the text that carries the information about the words penned by the author, and (ii) the cognitive operation that interprets these as a ‘story about a white whale.’

So, just as there is nothing in the physical text that is white or whale-like, there need be nothing in the physical brain that is phenomenally red. As Daniel Dennett has put it, the phenomenal quality of a purple sensation can be like ‘a beautiful discussion of purple, just about a colour, without itself being coloured’ [8, p. 371]. In which case, the task becomes to explain how the brain achieves this remarkable feat of representation: precisely how and why the internal ‘discussion of sensations’ takes the beautiful form it does.

## 6. **‘**Sentition**’**

My approach has been by ‘forward engineering’. That is to say, I have begun with the end product—sensations as humans experience them today—but, rather than treating this, as theorists typically do, as something to deconstruct, I have treated it as something to invent. I have tried to come up with an evolutionary sequence that can get us from nothingness to somethingness: from the blindsight of our remote ancestors to the fully phenomenal experience we humans enjoy today.

I suggested, just now, that when we have a sensation, it is rather as if we are expressing our bodily opinion about the sensory stimulus. I have taken that as the place to begin. I believe sensations did indeed originate as active behavioural responses to sensory stimuli: they were something the subject *did* about the stimulation long before they evolved to be something the subject *felt* about it.

So, I want you to imagine a primitive amoeba-like animal floating in the ancient seas. Stuff happens to it. Light falls on its body, pressure waves press against it, chemicals stick to it. Some of these events are going to be good for the animal, others bad. If it is to survive, it must evolve the ability to sort out the good from the bad, and to respond differently appropriately—reacting to this stimulus with an Ouch!, and to that with a Whoopee!

**Figure 1.**
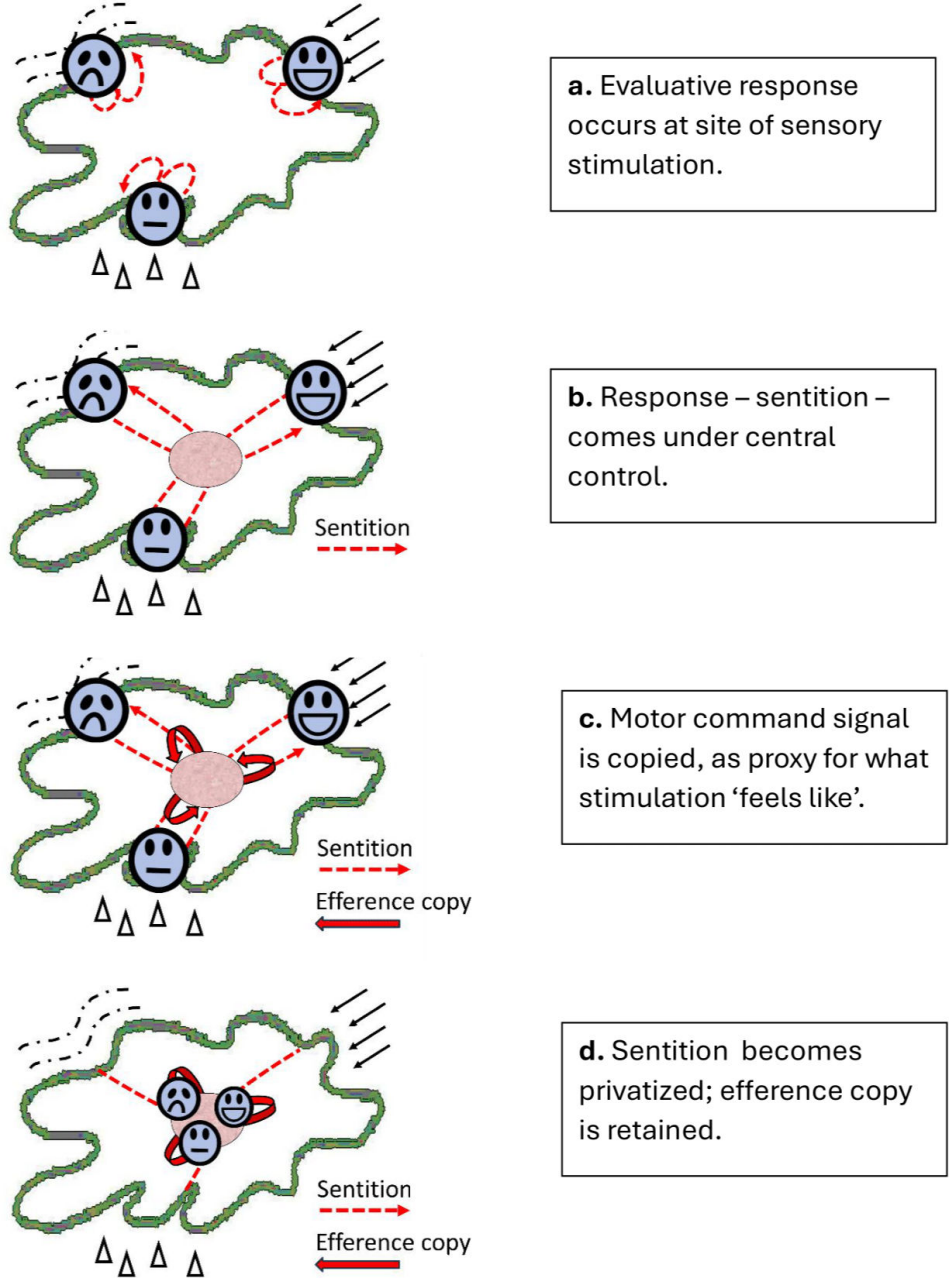
Evolution of sentition (earliest stages).

I call these expressive responses that evaluate the stimulation ‘sentition’ (a word combining sensation and volition). To start with, they are entirely local responses—wriggles of acceptance or rejection—organized around the site of the stimulus (figure 1a). When, say, red light arrives at the body surface, the animal makes a characteristic wriggle of activity—it wriggles ‘redly’. When salt arrives, it makes a different kind of wriggle—it wriggles ‘saltily’.

From the beginning, sentition has been designed by natural selection to be adaptive, with each response taking account of what kind of stimulus is reaching the body surface, and what importance it has for the animal’s well-being. Thus, even from the earliest stage, we could say the animal is *enacting what the stimulation means to it*. Yet, even after the responses become centralized (figure 1b), they remain entirely reflex and none of this meaning is being held ‘in mind’.

However, as the animal’s life becomes more complicated and demanding, it reaches a stage where it would benefit from retaining some kind of ‘mental record’ of what is affecting it: a representation of the stimulus that can serve as a basis for planning and decision-making. Now, as it turns out, there’s a neat way of achieving this. To discover ‘what’s happening to me’, the animal has only to monitor ‘what I’m doing about it’; and it can do this by the simple trick of creating a copy of the command signals for the responses—an ‘efference copy’ that can be read in reverse to recreate the meaning of the stimulation (figure 1c).^1^

In short, the animal can begin to get a feel for the stimulus by accessing the information already implicit in its own response. This, I believe, is the precursor of subjective sensation, although, of course, it will not at first be sensation as we humans know it: it will not have any special phenomenal quality.

## 7. Privatization and feedback

The key to acquiring phenomenal properties lies in how sentition goes on evolving. In the early days, it involves bodily behaviour, out in the open, but there must come a time when such overt behaviour is no longer appropriate. The animal no longer wants to recoil reflexly from red light, for example. However, it still wants to register that red light is falling on its body and that it feels menacing. So what to do?

The solution natural selection hits on is ingenious. It is for the responses to become internalized, or ‘privatized’. What happens is that the command signals, rather than bringing about actual motor behaviour, begin to target the internal body-map where the sense organs project to the brain (figure 1d). In this way, sentition evolves to be a *virtual* form of bodily expression —yet still an activity that can be read to provide a mental representation of the stimulation that elicits it.

**Figure 2.**
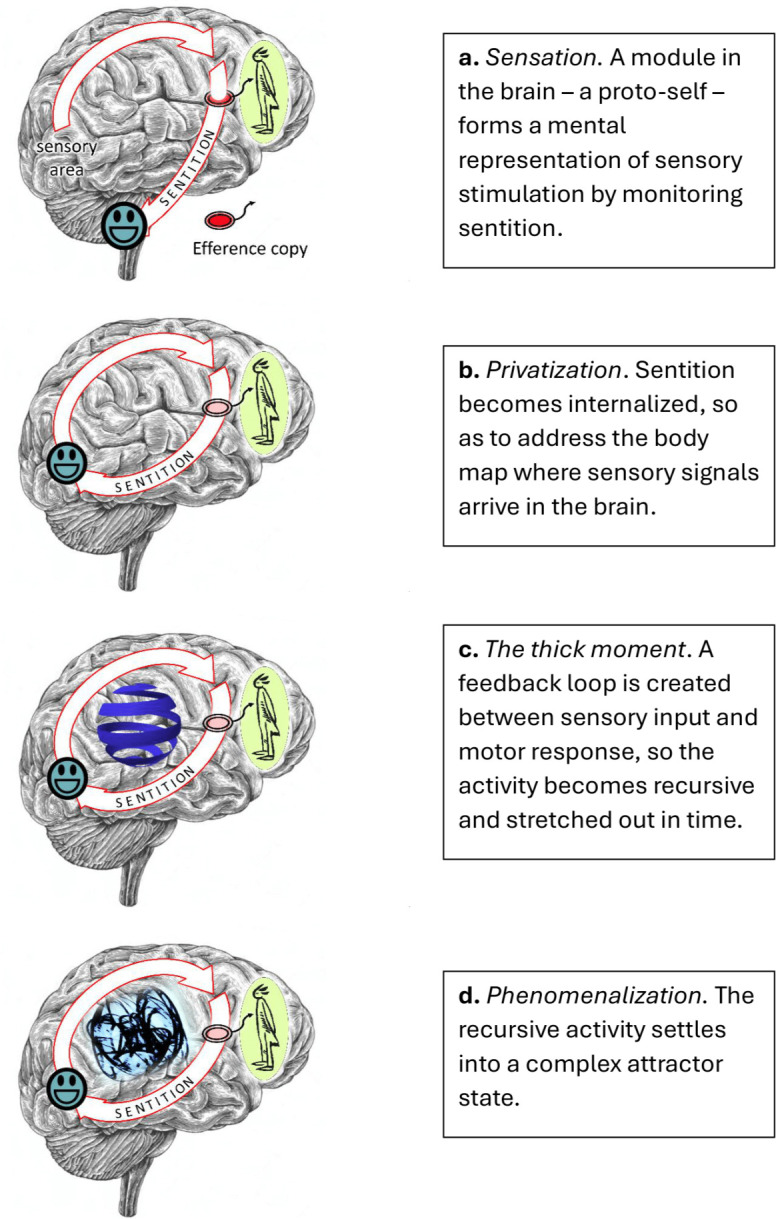
Evolution of sentition (later stages).

As luck would have it, this privatization has a wonderful result. To illustrate, let us switch to something more like a human brain (figure 2). Here we have sentition, the evaluative bodily response, being monitored by a proto-self—the new subject of sensation (figure 2a), and here the response is being privatized (figure 2b). The result is the creation of a feedback loop between motor and sensory regions of the brain—a loop with the potential to sustain recursive activity, going round and round, catching its own tail (figure 2c).

## 8. Attractors and the creation of ‘surreal’ properties

This feedback, I believe, is game-changing. Crucially, it means the activity can be drawn out in time, so as to give rise to the ‘thick moment’ of sensation. However, more than this, the activity can be channelled and stabilized, so as to create a mathematically complex ‘attractor’ state—a dynamical pattern of activity that recreates itself (in fact, a succession of such patterns).

Suppose the transmission characteristics of the loop are made to depend on the activity the previous time around. Then the development of the activity will be governed by a ‘delay differential equation’. Such feedback can, in principle, give rise to a family of complex attractors, with strange hyper-dimensional properties. This means that, from hereon in evolution, natural selection has a whole new design space to explore. Whenever it would be advantageous to ‘improve’ the quality of sensations, small adjustments to the circuitry can bring about dramatic changes to the attractor’s shape, with corresponding changes in the subjective experience.

I believe the upshot—in the line that led to humans and other animals that experience things as we do—has been the creation of a very special kind of attractor, which the subject reads as a sensation with the unaccountable feel of phenomenal *qualia*. This attractor is still a type of sentition, that originates as a response to sensory stimulation, and still carries valid information about ‘what is happening to me’. However, this information now comes in a remarkable new package. It comes, if you like, as part of a riddle written on the brain (see figure 2d).

Note that the representation of sensation that results is not an *illusion*. The subject is not being misinformed about what is happening. Rather, the brain is taking advantage of a ‘spare dimension’—a new degree of freedom—created by the feedback loops, to add what might be called a meta-physical commentary to the ‘discussion’ [10].

## 9. What is it for?

The sequence I have outlined is, I admit, something of a try-on. At best, it can be only partly right, but I submit that every step is plausible as an evolutionary development, and it leads to an end state that could in principle be responsible for sensory experience as we humans know it. I like to think it provides a proof of principle that a materialist explanation of phenomenal consciousness is possible.

I acknowledge, however, that the evolutionary explanation is seriously incomplete. For we still have to explain *what it’s all for*? What can have driven these historical developments, and those final stages in particular? What can have been the biological advantage to our ancestors of having sensations dressed up in this surreal way?

There are a good many theorists quite willing and even happy to answer: ‘None. Phenomenal consciousness isn’t *for* anything at all?’ To quote philosopher Jerry Fodor: ‘[phenomenal] consciousness ... seems to be among the chronically unemployed. . . As far as anybody knows, anything that our conscious minds can do they could do just as well if they weren’t conscious. . . Why then did God bother to make consciousness? What on earth could he have had in mind?’ [11, p. 31]. As I said, I want to leave God out of it. But we must still try to answer on behalf of natural selection.

In seeking the biological function of phenomenal consciousness, the place to start, surely, is with our own first-person intuitions. For who can know better than we ourselves do what *good* it does us to experience things the way we do? So, let us ask: what would be missing if we *lacked* phenomenal consciousness: if we had blindsight, blind-touch, blind-hearing, blind-everything?

I think there is an obvious and true answer, and it is the one I touched on when discussing blindsight. It is that what would be missing would be *us*.

As I remarked earlier, one of the most striking facts about human patients with blindsight is that they do not take ownership of their capacity to see. Lacking visual sensations with phenomenal properties—lacking the ‘somethingness’ of seeing—they believe that their manifest capacity for visual perception has *nothing to do with them*. Then, imagine what it would be like if we were to lack phenomenal experience of any kind at all: if we were to believe that none of our sensory experience was owned by us? Presumably, our ‘sense of self’ would disappear.

Of course I am not alone in saying this. The Scottish philosopher, David Hume, was in no doubt that conscious sensations provide the basis for selfhood (he writes of ‘perception’ rather than ‘sensation’, but the context makes it clear he means sensation):

For my part, when I enter most intimately into what I call myself, I always stumble on some particular perception or other, of heat or cold, light or shade, love or hatred, pain or pleasure. I never can catch myself at any time without a perception, and never can observe any thing but the perception. When my perceptions are remov’d for any time, as by sound sleep, so long am I insensible of myself, and may truly be said not to exist [12, p. 534].

So: remove phenomenal experience and the self ceases to exist. However, by the same token, install or reinstall it and the self leaps into existence! ^2^

## 10. The society of selves

Let us think back, then, to that point deep in the past when natural selection first breathed life into the loops in our ancestors’ brains and they woke up to find themselves transformed—into sentient self-conscious beings.

In reality, of course, it will not have happened in a flash, but I do not think it had to be a gradual process either. For the fact is that attractors of the kind I have invoked to explain it have an all-or-nothing character. So the phenomenalization of sensations could have come about quite rapidly, perhaps within a few hundred generations.

Whenever it occurred, it must certainly have been a psychological and social watershed. With this marvellous new phenomenon at the core of our being, we will each have begun to *matter* to ourselves in a new and deeper way. We will have come to believe, as never before, in our own singular significance, and it will not just have been each of us as individuals. For it will soon have dawned on us that other members of our species most likely possess conscious selves like ours. Thus, we will have been led to respect *their* individual worth as well. *‘I feel, therefore I am.’ . . ‘You feel, therefore you are too.’*

In fact, we will soon have made a life-changing discovery: it is that, when we imagine ourselves in our fellow creature’s place, we can model, *in ourselves*, what *they* are feeling. In short, phenomenal consciousness will have become our ticket to having a theory of mind and living in what I have called ‘the society of selves’.[14]

## 11. Warm-bloodedness, the game-changer?

If this account of how and why sentience has evolved in our own ancestors is even nearly right, it has inescapable implications for the distribution of phenomenal consciousness across the animal kingdom. Among the huge variety of animals alive today, which species are likely to have crossed the line?

There must be two crucial considerations. It is going to depend on the kind of brain the animal has, and the kind of life it leads. First, there will be no physiological means for generating phenomenal experience unless the animal has a brain that can house sensory-motor feedback loops capable of creating attractors of the kind I have described. Second, there will have been no evolutionary incentive for the animal to acquire these attractors unless it has a lifestyle in which possession of a phenomenally enriched sense of self can enhance its personal and social survival.

This leads me to a surprising—and possibly unwelcome—conclusion. I think sentience must be a rather recent evolutionary innovation. By far the majority of animals on Earth have neither the brains nor the use for it. To stick my neck out, I will be more specific: I suspect that sentience may not have arrived until the evolution of warm-blooded animals, mammals and birds, around 200 million years ago.

I draw the line there for two reasons. The evolution of warm-bloodedness had major effects both on animals’ lifestyles and on their brains.

For a start, it made animals relatively independent of environmental conditions. Cold-blooded animals not only have to stay within narrow geographical limits, but they have their activity levels dictated from moment to moment by the ambient temperature. As the sun sets—or goes behind a cloud—their bodies chill and their muscles and nerves slow down. By contrast, warm-blooded animals take their environment with them, and so can be alert and active—feeding, socializing, travelling—both by day and night, winter and summer, high in the mountains or down on the plains.

As warm-blooded animals became more self-reliant at a bodily level, they would have become more independent at a psychological level too. After millions of years in which their ancestors’ lives had been constrained by environmental temperature, they would have found themselves let off the leash, with the freedom to go where they would when they would. Suddenly, a huge constraint on selfhood had been lifted.

However, that is only half the story. For just at the time when warm-bloodedness was giving animals a new degree of bodily and psychological autonomy, it would also have been having a dramatic effect on their brains. The conduction speed of nerve cells increases with temperature by about 5% °C^−1^. This means that as body temperature increases from an average of say 15°C in cold-blooded animals to a steady 37°C in mammals and 40°C in birds, the speed of nerve cells in the brain will have nearly tripled. Because this will have reduced the time-lag in any feedback loops, it will have made recurrent activation all the more likely. I suggest this could have been just what was needed to fire up the attractor states on which the phenomenal self depends.

## 12. How does sentience show?

Now, where is the *evidence* about the reach of sentience? It is sometimes said we should not expect there to be any evidence because sentience is all on the inside and invisible to external observers. However, if sentience has evolved by natural selection, this cannot be true. There must have been effects that increase the animal’s chances of survival, and if selection has been able to see these effects in the past, presumably behavioural scientists should be able to do so today.

So where should scientists look? Given that phenomenal consciousness is an idea in the mind, presumably the effects must begin at the level of psychology. As I suggested above, I suspect the main effects will be on attitudes towards the self: first, about *what it’s like to be me,* and then, about *what it’s like to be you*.

I’ve recently proposed several ways in which this is likely to show up in behaviour. In my book, ‘*Sentience; the invention of consciousness*’ [15], I ask:

do animals:

(i) have a robust sense of self, centred on sensory experience?
(ii) engage in self-pleasuring activities? Sensations for sensation’s sake.
(iii) have notions of ‘I’ and ‘you’, as mirror selves?
(iv) carry their sense of their own identity forward?
(v) lend out their minds so as to understand others’ feelings?

I accept that none of these lines of evidence can seal the deal entirely, but they add together, and, while there is not space to expand on this here, I reckon the balance of evidence supports my hunch that it *is* only mammals and birds that make the cut. Chimpanzees, dogs, and parrots all affirm their selfhood in ways like those listed. Lobsters, lizards, frogs really do not.

Octopuses? They are everybody’s favourite candidate for an outlying species that is sentient. However, I think the behavioural evidence belies this. Octopuses are undoubtedly highly intelligent. Yet, on the face of it, they do not find pleasure in sensation-seeking; they do not have a strong sense of themselves as individuals; they do not attribute selfhood to others; nor do they care.

In short, I guess sentience would be wasted in octopuses. Suppose we could in fact genetically engineer an octopus to have phenomenal consciousness. I am pretty sure the new-found selfhood would make little or no difference to the octopus’s survival; so, the new genes would not be maintained by selection and would soon disappear.

## 13. The frog’s eye and the monkey’s brain

I want to return to Helen and blindsight. Many years ago, I wrote a paper titled ‘What the frog’s eye tells the monkey’s brain.’ [16] I think it’s now time to consider ‘What a monkey’s blindsight tells us about frogs.’

I would ask you to compare the cases of Helen reaching with her hand for peanuts and a bullfrog catching ants with its tongue (electronic supplementary material, video S2 shows them side by side). Both animals are having to use the subcortical visual pathways of their brains: Helen because the cortical system has been destroyed, the frog because it never had one. With blindsighted Helen, we must assume that, despite her obvious visual competence, there was ‘nothing it was like’ for her to see. I conclude that, equally, there is nothing it is like for the frog to see. Furthermore, there is probably nothing it is like for the frog to taste, or hear, or feel pain. I think the same goes for most animals on Earth.

However, I am not going to let the question rest there. For I have lately come round to thinking that if animals such as frogs are not phenomenally conscious, this does not necessarily mean that they are not conscious in any way at all. They could still be conscious in a more robotic, zombie-like way: still ‘cognitively conscious’, with subjective access to mental representations.

## 14. Cognitive consciousness

I will need to unpack this. I started this paper with Locke’s definition of consciousness as ‘perception of what passes in a man’s own mind’; and I homed in on sensations and phenomenal consciousness, as the most impressive feature. However, I should have acknowledged that cognitive scientists, who study how the mind works, have generally adopted Locke’s wider definition.

In cognitive science, consciousness is seen primarily as a technique for managing the flow of information by the brain. The best-known model of cognitive consciousness (which I am pretty sure is right) is Global Workspace Theory. This posits that there is a central processing unit in the brain that has access to a ‘global workspace’ where a selected set of mental representations are on display. The central processor, having an overview of the workspace, is able to collate and integrate information across different mental domains, so as to allow intelligent judgments and decisions to be made on behalf of the whole system. As such, cognitive consciousness is an effective *computational strategy*. It streamlines the work of the brain, resolves potential conflicts and gives coherence and direction to thoughts and actions.

That is *all* it does. There is no mention in this model of phenomenal quality. I do not think anyone would say it is cognitive consciousness that makes life worth living! Nonetheless, from a biological viewpoint, the advantages of cognitive consciousness are obvious. It gets results at the level of behaviour. It makes animals cleverer. What is more, it is a relatively simple brain process to engineer (man-made artificial intelligence already incorporates versions of it).

I am sure, therefore, that cognitive consciousness will have been discovered by natural selection and installed in animals’ brains very early on in evolution. This will have been long before phenomenal consciousness arrived in certain species as an extraordinary add-on.

To sum up, I now want to suggest there are actually three classes of animals on the spectrum of consciousness. ‘Unconscious’ (e.g. worms and jelly-fish), ‘cognitively conscious but NOT sentient’ (for example, bees, octopuses and frogs) and ‘cognitively conscious AND sentient’ (for example, parrots, dogs and humans).

## 15. Conscious blindsight?

However, where does this leave Helen? I assume Helen, like humans with blindsight, did not experience visual sensations as having a phenomenal dimension. Yet is it possible that she—like the frog and others of its class—could have been *cognitively conscious* of what she was seeing?

I have to say this possibility has only recently occurred to me, but I have been back over the films I made of Helen, and I have found tell-tale signs of her *dithering* mentally—apparently weighing up what course to follow. The electronic supplementary material, video S3 shows two examples: (i) she approaches an obstacle in her path, pauses, leans to the left, then to the right, before choosing to go the left, and (ii) she stares at a peanut that is just beyond her reach, prepares to extend her arm and then chooses to hold back. I think the word ‘chooses’ is the obvious epithet to describe her behaviour, but presumably Helen could only have *chosen* between alternatives if she were in fact cognitively conscious of them.

However, this raises a puzzling question about the difference with human blindsight. If Helen *knew* what she was seeing, why do humans with blindsight typically present as having little or no conscious access?

I think there is a plausible explanation for the difference. In almost all human cases, the fact is that the brain damage has been unilateral, leaving normal conscious vision in the intact part of the cortical visual field. With this imbalance, we might well expect the remaining normal vision to be psychologically dominant—which could get in the way of the patient recognizing that he does in fact still have cognitive access to percepts in the blind field.

This explanation is supported by a remarkable new case. Patient TN suffered two successive strokes that caused complete bilateral destruction of the visual cortex (V1), with relatively little further damage. At the invitation of neuropsychologist Bea de Gelder, I had the privilege of examining TN [17]. He claimed that he no longer had visual sensations *anywhere* in his visual field. However, he demonstrated a quite exceptional degree of blindsight, including the ability to navigate through a room full of obstacles, to recognize facial expressions, and to correctly identify the colours of objects. Yet, what is especially remarkable is that, quite unlike other cases, he was clearly aware of his visual percepts and would spontaneously comment on them without prompting (electronic supplementary material, video S4 shows examples of recognizing biological motion and colour).

New research on blindsight in monkeys by Takashi Isa and colleagues supports the idea that blindsight may be consciously accessible. As he writes, his experiments suggest that ‘higher-order cognitive functions such as working memory and associative learning can be achieved in blindsight’. [18, p. 1]

## 16. Atavistic vision

I would hesitate for obvious reasons to call blindsight, as it occurs in monkeys or humans, a case of evolutionary ‘regression’. However, it is a provocative thought: that blindsight is indeed a functional equivalent of the non-phenomenal vision of our pre-mammalian ancestors. In fact, I would go further and suggest that blindsight precedes fully phenomenal sight in human infancy.

The clue that humans at birth may lack phenomenal vision comes from anatomy. The fact is, babies are born before their cerebral cortex has fully developed. In the words of a recent paper: ‘contrary to the theory that V1 is myelinated at birth our analyses . . suggest that while V1 is more developed than other visual areas at birth, it continues to develop and profoundly myelinates during the first 6 months of life’ [19, p. 7].

This provides a compelling reason to believe that newborn babies at first rely on subcortical vision and in effect have blindsight. If that is so, it will only be as the brain becomes fully myelinated, and the cortical circuits begin to light up, that phenomenal vision emerges. Arguably, ‘ontogeny repeats phylogeny’.

Whether the same could be true across all sensory modalities is an open question. Contemporary philosophers tend to be dismissive of the possibility—taken seriously before now—that newborn human babies do not experience phenomenal pain [20]. However, many philosophers are equally sure that lobsters are phenomenally conscious [21]. In both cases, I would suggest the philosophical consensus may be wrong.

## Data Availability

Supplementary material is available online [22].

## Footnotes

^1^
The ‘efference copy’ of motor signals did not have to be newly created; it was very likely already in existence, having evolved early in the history of the brain as an essential component of the self-monitoring loop that enables an animal to set aside sensory stimulation that results from its own actions. See seminal discussion by Vallortigara [9].

^2^
While Hume does indeed emphasize sensory experience—‘heat or cold, light or shade'—he also includes emotional states—‘love or hatred, pain or pleasure’—that go beyond simple sensation. Without question, emotions can be felt, and so can play a role in anchoring the self, but do emotions have non-sensory phenomenal content? Or is it always a case of ‘borrowed phenomenality' that can be traced to somatic and visceral sensation—arising in the stomach, the chest, the face, the limbs, the heart? Expert commentators disagree, but I would take a strong line on this: sensations are the only class of mental representation that have phenomenal content—‘qualia’—such as we have been discussing in this paper. My one concession is that I allow for the existence ‘agentic qualia’ associated with preparation for bodily action [13].
